# TRAF2 Is a Novel Ubiquitin E3 Ligase for the Na,K-ATPase β-Subunit That Drives Alveolar Epithelial Dysfunction in Hypercapnia

**DOI:** 10.3389/fcell.2021.689983

**Published:** 2021-07-02

**Authors:** Nieves M. Gabrielli, Luciana C. Mazzocchi, Vitalii Kryvenko, Khodr Tello, Susanne Herold, Rory E. Morty, Friedrich Grimminger, Laura A. Dada, Werner Seeger, Jacob I. Sznajder, István Vadász

**Affiliations:** ^1^Member of the German Center for Lung Research (DZL), Department of Internal Medicine, Justus Liebig University Giessen, Universities of Giessen and Marburg Lung Center (UGMLC), Giessen, Germany; ^2^The Cardio-Pulmonary Institute (CPI), Giessen, Germany; ^3^Department of Lung Development and Remodeling, Max Planck Institute for Heart and Lung Research, Bad Nauheim, Germany; ^4^Institute for Lung Health (ILH), Giessen, Germany; ^5^Division of Pulmonary and Critical Care Medicine, Feinberg School of Medicine, Northwestern University, Chicago, IL, United States

**Keywords:** Na, K-ATPase β-subunit, carbon dioxide, ubiquitination, endocytosis, PKC-ζ, TRAF2, hypercapnia, adherens junction

## Abstract

Several acute and chronic lung diseases are associated with alveolar hypoventilation leading to accumulation of CO_2_ (hypercapnia). The β-subunit of the Na,K-ATPase plays a pivotal role in maintaining epithelial integrity by functioning as a cell adhesion molecule and regulating cell surface stability of the catalytic α-subunit of the transporter, thereby, maintaining optimal alveolar fluid balance. Here, we identified the E3 ubiquitin ligase for the Na,K-ATPase β-subunit, which promoted polyubiquitination, subsequent endocytosis and proteasomal degradation of the protein upon exposure of alveolar epithelial cells to elevated CO_2_ levels, thus impairing alveolar integrity. Ubiquitination of the Na,K-ATPase β-subunit required lysine 5 and 7 and mutating these residues (but not other lysines) prevented trafficking of Na,K-ATPase from the plasma membrane and stabilized the protein upon hypercapnia. Furthermore, ubiquitination of the Na,K-ATPase β-subunit was dependent on prior phosphorylation at serine 11 by protein kinase C (PKC)-ζ. Using a protein microarray, we identified the tumor necrosis factor receptor-associated factor 2 (TRAF2) as the E3 ligase driving ubiquitination of the Na,K-ATPase β-subunit upon hypercapnia. Of note, prevention of Na,K-ATPase β-subunit ubiquitination was necessary and sufficient to restore the formation of cell-cell junctions under hypercapnic conditions. These results suggest that a hypercapnic environment in the lung may lead to persistent epithelial dysfunction in affected patients. As such, the identification of the E3 ligase for the Na,K-ATPase may provide a novel therapeutic target, to be employed in patients with acute or chronic hypercapnic respiratory failure, aiming to restore alveolar epithelial integrity.

## Introduction

The Na,K-ATPase heterodimer, composed of a catalytic α- and a regulatory β-subunit, is expressed in all animal tissues, establishing concentration gradients for Na^+^ and K^+^ by pumping three Na^+^ ions out of the cell and two K^+^ ions into the cell in an ATP-dependent manner (Blanco et al., 1994; Kaplan, 2002). Apart from its well-established role in vectorial sodium transport, the Na,K-ATPase is critical for maintaining epithelial barrier integrity (Rajasekaran et al., 2005; Cereijido et al., 2012; Vagin et al., 2012). The Na,K-ATPase co-localizes with adherens junctions in all stages of monolayer formation, and the Na,K-ATPase β-subunit acts as a cell adhesion molecule that organizes and maintains the integrity of intercellular junctions (Vagin et al., 2006; Vadasz et al., 2007; Tokhtaeva et al., 2011).

In patients with severe lung diseases such as the acute respiratory distress syndrome (ARDS), the alveolar epithelium is injured and cell junctions are disrupted, resulting in accumulation of alveolar edema fluid and impaired gas exchange (Ware, 2006; Herold et al., 2013). As a consequence, patients with ARDS often present with elevated levels of CO_2_ (hypercapnia), a condition that may be further exacerbated by mechanical ventilation with low tidal volumes by the use of the so-called “permissive hypercapnia” strategy that aims to limit further ventilator-induced lung injury (Vadasz et al., 2012b; Herold et al., 2013). A key factor in patient recovery is the reconstitution of a normal alveolar structure, which involves the re-organization of cell junctions (Crosby and Waters, 2010). However, it remains unknown whether the formation of new cell-cell contacts is affected by a hypercapnic environment. We have previously demonstrated that hypercapnia impairs alveolar fluid reabsorption by inducing the endocytosis of the Na,K-ATPase α-subunit from the cell surface (Briva et al., 2007; Vadasz et al., 2008, 2012b; Vadasz and Sznajder, 2017). In the present study, we investigated whether hypercapnia affects the stability of the Na,K-ATPase β-subunit at the plasma membrane, thereby negatively impacting formation of cell-cell contacts.

The ubiquitin system has emerged as a central mechanism implicated in the regulation of protein subcellular localization. Apart from the canonical role of ubiquitination in mediating protein degradation, ubiquitination has been shown to be required for internalization and sorting of several plasma membrane proteins (Hicke, 1997; Hershko and Ciechanover, 1998; Gwozdzinska et al., 2017). The ubiquitination reaction occurs *via* sequential action of three enzymes: a ubiquitin-activating enzyme E1, a ubiquitin-conjugating enzyme E2 and a ubiquitin ligase E3 (Hershko and Ciechanover, 1998). The ubiquitin E3 ligases determine the specificity of the ubiquitination reaction by specifically recognizing their substrates, and therefore, may be considered as potential therapeutic targets (Metzger et al., 2012).

Here we provide novel insights into the hypercapnia-induced dysfunction of the alveolar epithelium. We demonstrate that hypercapnia impairs cell-cell contact formation by reducing the protein levels of the Na,K-ATPase β-subunit at the plasma membrane. Furthermore, we show that elevated CO_2_ leads to PKC-ζ-mediated regulation of Na,K-ATPase β-subunit, which triggers ubiquitination of the protein, resulting in subsequent endocytosis and degradation of the Na,K-ATPase β-subunit. Most importantly, using a protein microarray, we identify TRAF2, a RING E3 ligase probably best known for the role in the regulation of the NF-κB signaling pathway (Xia and Chen, 2005), as the key player promoting ubiquitination of the Na,K-ATPase β-subunit. Since E3 ligases impart specificity to the ubiquitination process, identifying the ubiquitin E3 ligase responsible for hypercapnia-induced ubiquitination of the Na,K-ATPase β-subunit may provide us with a highly specific tool that could be employed in therapies that aim to restore alveolar epithelial integrity, in patients with severe lung diseases such as acute respiratory distress syndrome, lung cancer, and chronic obstructive pulmonary disease.

## Materials and Methods

### Reagents, Plasmids, and siRNA

The antibodies used were as follows: rabbit anti-E-cadherin (H-108), mouse anti-ubiquitin (clone P4D1), mouse anti-PKC-ζ (H-1) and rabbit anti-TRAF2 (C-20) from Santa Cruz Biotechnology; mouse anti-Na,K-ATPase β1-subunit (clone M17-P5-F11), HRP-conjugated goat anti-mouse IgG and FITC-conjugated rabbit anti-mouse IgG from Thermo Scientific; rabbit anti-V5 and rabbit anti-actin from Sigma-Aldrich; Alexa Fluor 647-conjugated mouse anti-V5 antibody and mouse anti-V5 from Invitrogen; mouse anti-HA (clone 16B12) from Covance; HRP-conjugated goat anti-rabbit IgG were from Cell Signaling. Mouse anti-GFP (clones 7.1 and 13.1) were from Roche Diagnostic. MG-132 was purchased from Calbiochem. Chloroquine, cycloheximide (CHX) and N-Ethylmaleimide (NEM) were purchased from Sigma-Aldrich. Bisindolylmaleimide I, Hydrochloride was from Cell Signaling. siRNA against PKC-ζ was from cell-signaling and siRNA against TRAF2 and control siRNA-A were from Santa Cruz Biotechnology. Lipofectamine 2000, lipofectamine RNAiMax Reagent and Opti-MEM I reduced serum medium were from Invitrogen. Bradford reagent was from Bio-Rad. Halt Protease and Phosphatase Inhibitor Cocktail was purchased from Thermo Scientist. EZ-link NHS-SS-biotin and high capacity streptavidin agarose beads were from Pierce Biotechnology. Vectashield mounting medium was from Vector Laboratories. All biotinylated synthetic peptides were purchased from BIOMATIK and are indicated in Supplementary Table 1. The ubiquitin protein microarray was from LifeSensors (MA101). Human recombinant ubiquitin, UBE1, UbcH13/Uev1a, ubiquitin aldehyde, 10 × ubiquitination reaction buffer, 10 × ATP/Mg^+^ were from Boston Biochem. Sphingosine-1-phosphate was from Avanti Polar Lipids. The mRNA isolation kit and the plasmid purification kit were from Qiagen. JM109 competent bacteria were obtained from Promega. Reagents for production of cDNA and PCR were from Bio-Rad. Restriction endonucleases were obtained from Thermo Fisher Scientific. DNA ligase was from Promega. pcDNA 3.1 V5-His was purchased from Invitrogen. The cDNA encoding human Na,K-ATPase β1-subunit was amplified from A549 cells by PCR and cloned in between BAMHI and ECORI of the vector pcDNA 3.1 V5-His (A) for mammalian expression. Protein expression of recombinant V5-tagged Na,K-ATPase-β1 (V5-β1) is shown in Supplementary Figure 1A. V5-β1 gives a very strong antibody signal and upon short exposure of these blots to x-ray films, the signal appears as a single band. However, upon longer exposure, the same band pattern is obvious as in the case of the endogenous protein, as a consequence of the glycosylation pattern of the protein (Supplementary Figure 1B). The Na,K-ATPase β1-subunit variants K5R, K7R, K5/7R, K13/14R; K21/22R, S11A and S11D were generated by site-directed mutagenesis with the Quick Change Mutagenesis Kit which was purchased in Stratagene. All primers were synthesized by METABION and are indicated in Supplementary Table 2. All PCR products were confirmed by DNA sequencing. HA-ubiquitin-wt (plasmid 17608) and pEBG-TRAF2-GST (plasmid 21586) were obtained from Addgene (Chadee et al., 2002; Lim et al., 2005).

### Isolation and Culture of AEC

Isolation of alveolar epithelial cells (AEC) from rats was approved by the local authorities (Regierungspräsidium Giessen) in Germany. Primary type II AEC were isolated from the lungs of pathogen-free male Sprague-Dawley rats weighing 200–225 g, as previously described (Ridge et al., 2003). The day of isolation and plating was designated culture day 0. All experiments were conducted on day 3. A549 cells (American Type Culture Collection CCL-185) were grown in DMEM high glucose (PAA Laboratories) containing 10% (v/v) fetal bovine serum (PAA Laboratories), 100 U/ml penicillin and 100 μg/ml streptomycin (both from PAN-Biotech). A549 cells stably expressing dog Na,K-ATPase β-subunit fused to yellow fluorescence protein (A549-β1-YFP) were cultured in similar conditions. Cells were incubated in a humidified atmosphere of 5% CO_2_ and 95% air at 37°C.

### CO_2_ Media and CO_2_ Exposure

For different experimental conditions, initial solutions were prepared with DMEM, Ham’s F12 medium, Tris base (3:1:0.5) supplemented with 10% fetal bovine serum. The buffering capacity of the media was adjusted by changing its initial pH with Tris base in order to obtain a pH of 7.4 at levels of CO_2_ of 40 mmHg (“Ctrl”) and 110 mmHg (“CO_2_”). The desired CO_2_ and pH were achieved by equilibrating media overnight in a humidified chamber (C-Chamber, BioSpherix Ltd.). The atmosphere of the C-Chamber was controlled with a PROCO_2_ carbon dioxide controller (BioSpherix Ltd.). In this chamber, cells were exposed to the desired pCO_2_ while maintaining 21% O_2_ balanced with N_2_. Prior to and after CO_2_ exposure, pH, pCO_2_, and pO_2_ levels in the media were measured using a blood gas analyzer (Rapidlab, Siemens). Experiments were started by replacing culture media with the CO_2_-equilibrated media and incubating in the C-Chamber for the desired time.

### Transfection of AEC

Transfection of A549 cells with DNA plasmids or siRNAs were done by using lipofectamine 2000 or lipofectamine RNAiMax Reagent, respectively, following the recommendations of the manufacturer. For cell aggregation experiments, nucleofection of A549 and primary rat ATII cells with DNA plasmid was conducted following a protocol recently described by our group (Grzesik et al., 2013). For primary rat ATII cells, P3 primary cell Nucleofector solution was employed; and for A549 cells, a cell-line Nucleofector solution was used (Lonza Company).

### Biotinylation of Cell Surface Proteins

Cell surface biotinylation was performed with EZ-link NHS-SS-biotin (Pierce Biotechnology) and the pull-down of biotinylated proteins was done with streptavidin-Sepharose beads (Thermo Scientific) following protocols which have been described in detail previously (Dada et al., 2003; Vadasz et al., 2008; Lecuona et al., 2009). For pulse-chase experiments, after quenching unreacted biotin with 100 mM glycine, warm and equilibrated media with normal or elevated CO_2_ levels was added to the cells. Then, after incubating at 37°C for the desired times, cells were washed and lysed in m-RIPA, and biotinylated proteins were isolated as previously indicated. Proteins were analyzed by SDS-PAGE and Western immunoblot.

### Cell Lysis and *in vivo* Protein Ubiquitination Detection

After experimental treatments, cells were lysed in modified radioimmunoprecipitation buffer (m-RIPA) consisting of 50 mM Tris–HCl, pH 8, 150 mM NaCl, 1% NP-40, 1% sodium deoxycholate supplemented with phosphatase/protease inhibitor cocktail and 12.5 μg/ml NEM as previously described (Dada et al., 2003; Vadasz et al., 2008; Lecuona et al., 2009). For detection of protein ubiquitination, 1% Triton X-100 and 0.1% SDS were added to 800–1,000 μg of protein lysate in 500 μl final volume. After preclearing with protein A/G PLUS Agarose (Santa Cruz Biotechnology), protein extracts were rotated with the indicated antibodies for 1 h at 4°C. Then antigen-antibody complexes were immunoprecipitated by adding 50 μl of protein A/G and rotated overnight at 4°C. Immunoprecipitates were washed 5 × with m-RIPA buffer containing protease inhibitors. Agarose beads were resuspended in 2 × Laemmli sample buffer solution (Laemmli, 1970) containing 20% β-mercaptoethanol and boiled for 10 min. Proteins were analyzed by SDS-PAGE and IB. For detection of plasma membrane protein ubiquitination, after labeling cell surface proteins with EZ-link NHS-SS-biotin, cells were lysed in m-RIPA buffer and protein immunoprecipitation was done as described above. Immunoprecipitates were thoroughly washed with m-RIPA and boiled for 5 min. in 30 μl of a buffer containing 25 mM Tris HCl pH 6.8 and 1% SDS supplemented with NEM and phosphatase/protease inhibitor cocktail to release immunoprecipitated proteins. Biotinylated proteins were isolated with streptavidin-sepharose beads as described above.

### Co-immunoprecipitation and *in vitro* Protein Interaction Studies

For co-immunoprecipitation experiments, cell were lysed by incubating 15 min in the presence of co-immunoprecipitation buffer containing 20 mM HEPES pH 7,4, 150 mM NaCl, 0.5% NP-40, 2 mM EDTA, 2 mM EGTA, 5% glycerol, protease/phosphatase inhibitor cocktail. Protein immunoprecipitation was conducted following the protocol stated above, using 1–3 mg of cell lysate. For *in vitro* protein interaction studies, cells were lysed with co-immunoprecipitation buffer and incubated with the indicated biotinylated synthetic peptides overnight. Biotinylated peptides were isolated with streptavidin-Sepharose beads as described above. Proteins were analyzed by SDS-PAGE and IB.

### *In vitro* Protein Ubiquitination

Endogenous TRAF2 was purified from A549 cells by immunoprecipitation in co-immunoprecipitation buffer supplemented with 2 mM DTT, 0.5% Triton X-100, 12.5 μg/ml NEM, 5 μM ubiquitin aldehyde and protease/phosphatase inhibitor cocktail. Ubiquitination reactions were carried out at 37°C for 2 h in 1× ubiquitin reaction buffer, 1× ATP/Mg^+^, 100 nM S1P, 50 nM E1 UBE1, 150 nM E2 UbcH13/Uev1a, 100 μg/ml human recombinant ubiquitin, A/G-agarose beads containing immunoprecipitated TRAF2 and biotinylated cyt-β1 wt peptide. Supernatants were recovered and resuspended in a buffer containing 20 mM Tris/HCl pH8, 100 mM NaCl, 1 mM EDTA, 0.5% NP-40, 5 μM ubiquitin aldehyde, protease/phosphatase inhibitor cocktail and biotinylated peptides were isolated with streptavidin-Sepharose beads as described above. Proteins were analyzed by SDS-PAGE and IB.

### Protein Quantification, SDS-PAGE and Western Immunoblotting

Protein concentration was quantified by the Bradford assay (Bio-Rad) according to the manufacturer instructions. Proteins were resolved on 10–15% polyacrylamide gels and transferred to nitrocellulose membranes (Optitran, Schleicher & Schuell) using a semidry transfer apparatus (Bio-Rad). Incubation with specific antibodies was performed overnight at 4°C. Blots were developed with a SuperSignal West Pico Chemiluminescent Substrate detection kit (Thermo Scientific), as recommended by the manufacturer. The bands were quantified by densitometric scanning (ImageJ).

### Immunofluorescence

A549 cells were plated on cover slips and prepared for fluorescence microscopy. After cell treatments, cells were fixed with 4% paraformaldehyde (Thermo Scientific, Rockford, IL, United States) for 15 min. After washing with PBS, cells were permeabilized by incubation with 0.1% Triton X-100 for 5 min and blocked with 3% BSA solution for 30 min. Immunostaining of the Na,K-ATPase β-subunit was performed by overnight incubation with antibodies against Na,K-ATPase-β followed by 1 h incubation with Alexa488-conjugated secondary antibody (Invitrogen, Rockford, IL, United States). Afterward, staining with phalloidin (Thermo Scientific, Rockford, IL, United States) was done for visualizing cellular plasma membrane. Finally, nuclei staining was performed by Hoechst 33342 (Thermo Scientific, Rockford, IL, United States). Images were captured by using the Leica TCS SP5 (Leica Microsystems, Wetzlar, Germany) confocal microscope.

### Cell Aggregation Assays

Cell aggregation was determined by using the hanging-drop assay as described previously (Kundu et al., 2008; Matos et al., 2017). Briefly, A549 cells or rat primary type II AEC (40,000 cells/drop; 4 drops/experiment) were transfected with V5-β1 wt and variants and exposed to normocapnia or hypercapnia during 4 or 8 h as indicated in figure legends, after which the whole drop was fixed with 3.7% formaldehyde and mounted. Pictures were taken and the number of cells per aggregate was determined. ImageJ was used for the analysis of the pictures. A scale was set in which the area corresponding to one average-size cell was call 1 unit, thus area units were equivalent to number of cells.

### Statistical Analysis

Values are reported as mean ± SEM. Statistical analysis was performed with Student’s *t* test or one-way ANOVA followed by Tukey’s *post hoc* test for multiple comparisons. A *p* value < 0.05 was considered significant.

## Results and Discussion

### Hypercapnia Induces Endocytosis and Proteasome-Mediated Degradation of the Na,K-ATPase β-Subunit in Alveolar Epithelial Cells

**FIGURE 1:**
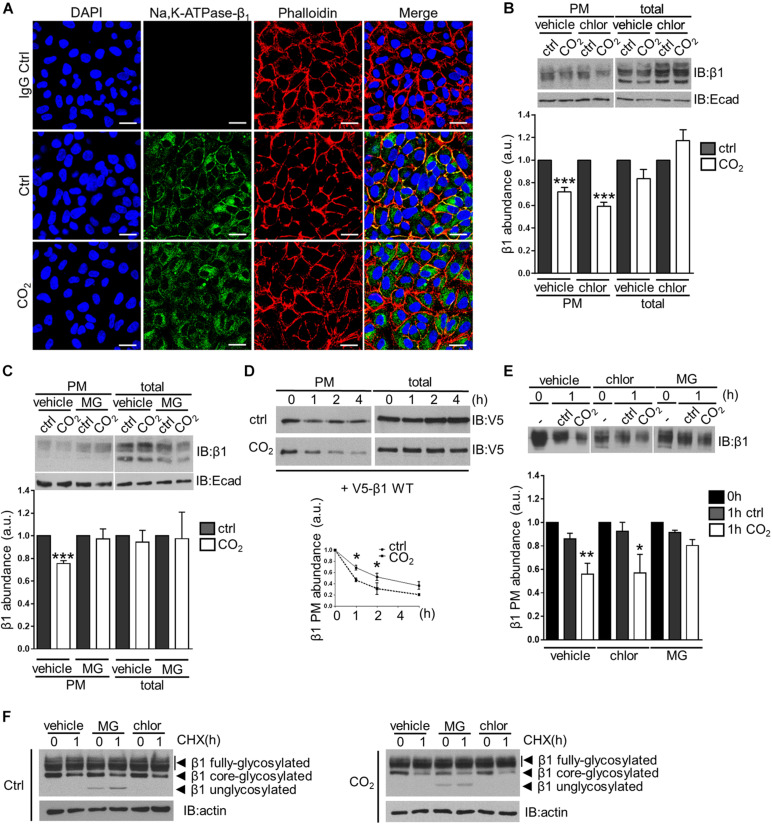
Elevated CO_2_ levels induce endocytosis and subsequent degradation of the Na,K-ATPase β-subunit in alveolar epithelial cells. **(A)** A549 cells were exposed to 40 or 110 mmHg CO_2_ at a pH of 7.4 for 30 min. Cellular localization of the Na,K-ATPase β-subunit was detected by confocal microscopy using an anti-Na,K-ATPase β1-subunit antibody (green) under normocapnia (Ctrl) or hypercapnia (CO_2_) and in the absence of the specific antibody (IgG Ctrl). Additional phalloidin (red) and nuclei (blue) staining are shown. Scale bar = 20 μM. **(B)** A549 cells were exposed to 40 or 110 mmHg CO_2_ at a pH of 7.4 for 30 min in the presence of vehicle (water) or chloroquine (100 μM). Na,K-ATPase β-subunit at the surface was determined by biotin-streptavidin pull-down and IB. Total protein abundance is also shown. Mean ± SEM, *n* = 3, paired *t*-test, ***p < 0.0005. **(C)** A549 cells were exposed to 40 or 110 mmHg CO_2_ at a pH of 7.4 for 30 min in the presence of vehicle (DMSO) or MG-132 (20 μM). Na,K-ATPase β-subunit at the surface was determined by biotin-streptavidin pull-down and IB. Total protein abundance is also shown. Mean ± SEM, *n* = 3, paired *t*-test, ***p < 0.0005. **(D)** A549 cells were transfected with V5-β1 wt and surface proteins were labeled with biotin and chased in 40 or 110 mmHg CO_2_ at a pH of 7.4 for different time points. Afterward, streptavidin pull-down was performed and Na,K-ATPase β-subunit abundance was analyzed by IB. Mean ± SEM, *n* = 3, paired *t*-test, *p < 0.05. **(E)** Surface proteins of A549 cells were labeled with biotin and chased in 40 or 110 mmHg CO_2_ at a pH of 7.4 for 1 h in the presence of vehicle (DMSO), chloroquine (100 μM), MG-132 (20 μM). Afterward, streptavidin pull-down was performed and Na,K-ATPase β-subunit abundance was analyzed by IB. Mean ± SEM, *n* = 3, paired *t*-test, *p < 0.05. **(F)** A549 cells were exposed to 110 mmHg CO_2_ at a pH of 7.4 for 1 h with CHX (1.7 mM) in the presence of vehicle (DMSO), chloroquine (100 μM), MG-132 (20 μM). Total Na,K-ATPase β-subunit abundance was determined by IB.

The transmembrane Na,K-ATPase β-subunit consists of 2 N-glycosylated forms that are detected in western blots as a broad band at 45–55 kDa that corresponds to the fully-glycosylated form, and a sharper band at ∼40 kDa, that corresponds to the core-glycosylated isoform. Only the mature, fully-glycosylated form is found in the plasma membrane of cells and mediates homophilic cell-cell adhesion. To assess the effects of hypercapnia on the stability of the Na,K-ATPase β-subunit at the cell surface, we conducted immunofluorescence studies in which A549 cells were exposed to elevated CO_2_ levels for 30 min. We observed a decrease in the Na,K-ATPase β-subunit abundance in the plasma membrane, accompanied by translocation of the protein into intracellular compartments (Figure 1A). In agreement with this observation, biotin-streptavidin pull-downs revealed a 30% reduction in Na,K-ATPase β-subunit abundance in the plasma membrane of cells exposed to hypercapnia compared to cells exposed to normocapnia. In contrast, total levels of the Na,K-ATPase β-subunit did not exhibit changes after treatment with elevated CO_2_ concentrations. Pre-treatment of cells with chloroquine, which results in the inhibition of the lysosomal function, did not prevent hypercapnia-induced endocytosis of the Na,K-ATPase β-subunit (Figure 1B). In contrast, pre-incubation of cells with the proteasome inhibitor MG-132 abrogated the hypercapnia-induced endocytosis of the Na,K-ATPase β-subunit (Figure 1C). To determine the fate of the endocytosed Na,K-ATPase β-subunit, plasma membrane proteins were labeled with cell-impermeable biotin and chased under normocapnic and hypercapnic conditions for up to 4 h. We observed that hypercapnia promoted the degradation of the transiently-expressed V5-tagged, wild-type Na,K-ATPase β1-subunit (V5-β1 wt), leading to a marked decrease in the half-life under hypercapnic conditions (Figure 1D). This effect was also observed for the endogenous Na,K-ATPase β-subunit, approximately 50% of which was degraded after 1 h of hypercapnia, as opposed to a markedly slower degradation rate under normocapnic conditions. In line with our previous findings, pre-incubation of cells with MG-132, but not with chloroquine, prevented the hypercapnia-induced degradation of the Na,K-ATPase β-subunit located in the plasma membrane (Figure 1E). Together, these studies demonstrate that exposure of cells to elevated CO_2_ levels leads to the endocytosis and degradation of the Na,K-ATPase β-subunit. Previous studies conducted by Yoshimura et al. (2008) in HeLa cells revealed that the degradation of the plasma membrane Na,K-ATPase β-subunit is prevented by proteasome inhibitors. Similarly, our results suggest that proteasomal activity was required for hypercapnia-induced degradation of the Na,K-ATPase β-subunit.

It is known that proteasome inhibitors have an impact on the degradation of various intracellular proteins, which may directly or indirectly affect Na,K-ATPase β-subunit stability. For instance, endocytic machinery proteins, kinases and E3 ligases may be regulated by the proteasome (Lee et al., 1996; Smith et al., 2000; Li et al., 2002). Moreover, free ubiquitin molecules are normally recycled after protein degradation by the proteasome (Hershko et al., 1984). Therefore, proteasome inhibition may eventually lead to ubiquitin depletion that may affect ubiquitin-mediated endocytosis signaling and lysosomal degradation (Carter et al., 2004; Melikova et al., 2006). However, as lysosomal inhibitors did not affect the Na,K-ATPase β-subunit stability, but inhibition of the proteasome did, we believe that the proteasome is involved in the degradation of Na,K-ATPase β-subunit under hypercapnic conditions. This finding is particularly interesting considering that degradation of plasma membrane proteins is generally lysosome-mediated (Piper and Luzio, 2007).

In another set of experiments, using the protein synthesis inhibitor cycloheximide (CHX), we were able to determine the stability of the endoplasmic reticulum (ER)-localized core-glycosylated form of the Na,K-ATPase β-subunit. After 1-h treatment with CHX, we observed a decrease in the protein abundance of this ∼40 kDa form of approximately 50% under both normocapnic and hypercapnic conditions. Interestingly, pre-incubation of cells with MG-132 did not prevent the decrease in the abundance of the core-glycosylated form of the Na,K-ATPase β-subunit, but led to the accumulation of a ∼35 kDa form, which corresponds to a unglycosylated form of the Na,K-ATPase β-subunit. In contrast, pre-treatment of cells with chloroquine did not have any effect (Figure 1F). These results are consistent with a model in which the ER-localized Na,K-ATPase β-subunit form undergoes ER-associated degradation (ERAD) in which glycoproteins are retro-translocated from the ER membrane, deglycosylated and degraded by the proteasome (Tsai et al., 2002; Meusser et al., 2005; Leichner et al., 2009; Kryvenko et al., 2020; Kryvenko and Vadasz, 2021).

### Hypercapnia-Induced Ubiquitination of the Na,K-ATPase β-Subunit Leads to the Endocytosis of the Protein

**FIGURE 2:**
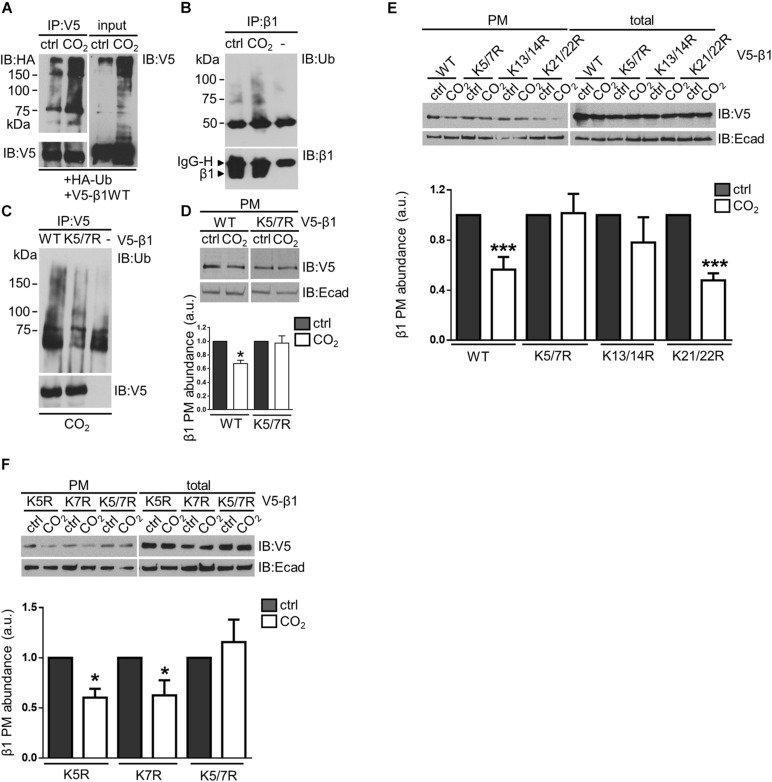
Ubiquitination of the Na,K-ATPase β-subunit acts as a signal for endocytosis of the protein at elevated CO_2_ levels in alveolar epithelial cells. **(A)** A549 cells were co-transfected with HA-ubiquitin (Ha-Ub) and V5- β1 wt. 18 h after transfection, cells were exposed to 40 or 110 mmHg CO_2_ at a pH of 7.4 for 15 min in the presence of MG-132. V5-β1 wt was immunoprecipitated with anti-V5 polyclonal antibody (IP) and ubiquitinated forms were detected with an anti-HA monoclonal antibody by IB. Loading was controlled with anti-V5 monoclonal antibody. Na,K-ATPase β-subunit polyubiquitinated forms were also detected in input with anti-V5 monoclonal antibody by overexposing the films. **(B)** A549 cells were exposed to 40 or 110 mmHg CO_2_ at a pH of 7.4 for 15 min. Biotin-streptavidin pull-down was performed to enrich in surface proteins. Na,K-ATPase β-subunit was IP with anti-β1-subunit monoclonal antibody and Na,K-ATPase β-subunit polyubiquitinated forms were detected with anti-ubiquitin monoclonal antibody by IB. **(C)** A549 cells were transfected with V5-β1 wt, V5-β1 K5/7R or empty plasmid. Cells were exposed to 40 or 110 mmHg CO_2_ at a pH of 7.4 for 15 min. V5-β1 wt or K5/7R were IP with anti-V5 polyclonal antibody and ubiquitinated forms were detected with anti-ubiquitin monoclonal antibody by IB. Loadings were detected with anti-V5 monoclonal antibody. **(D)** A549 cells were transfected with V5-β1 wt or V5-β1 K5/7R. Cells were exposed to 40 or 110 mmHg CO_2_ at a pH of 7.4 for 30 min. Biotin-streptavidin pull-downs were performed and V5-β1 wt or V5-β1 K5/7R abundance was analyzed by IB. Mean ± SEM, *n* = 3, paired *t*-test, *p < 0.05. **(E)** A549 cells were transfected with V5-β1 wt, V5-β1 K5/7R, V5-β1 K13/14R, V5-β1 K21/22R. Cells were exposed to 40 or 110 mmHg CO_2_ at a pH of 7.4 for 30 min. Biotin-streptavidin pull-downs were performed and V5-β1 abundance was analyzed by IB. **(F)** A549 cells were transfected with V5-β1 K5R, V5-β1 K7R or V5-β1 K5/7R. Cells were exposed to 40 or 110 mmHg CO_2_ at a pH of 7.4 for 30 min. Biotin-streptavidin pull-downs were performed and V5-β1 abundance was analyzed by IB.

Ubiquitin is a key player in the regulation of endocytosis of several membrane proteins (Hicke, 1997; Haglund and Dikic, 2012) and ubiquitination during normal turnover of the of the Na,K-ATPase β-subunit has been reported (Yoshimura et al., 2008). To evaluate whether ubiquitination played a role in the hypercapnia-induced endocytosis of the Na,K-ATPase β-subunit, A549 cells transfected with HA-ubiquitin and V5-β1 wt were exposed to normocapnia or hypercapnia for 15 min. V5-β1 wt was immunoprecipitated and the ubiquitinated Na,K-ATPase β-subunit forms were detected with an anti-HA antibody. We observed that hypercapnia promoted the ubiquitination of Na,K-ATPase β-subunit, detected as a smear characteristic of poly-ubiquitinated proteins, which was also evident in total protein lysates (input) (Figure 2A). To evaluate Na,K-ATPase β-subunit ubiquitination at the plasma membrane, we immunoprecipitated the Na,K-ATPase β-subunit from a pool of plasma membrane proteins and detected the ubiquitinated Na,K-ATPase β-subunit forms with an anti-ubiquitin antibody. The experiments revealed a significant increase in the abundance of ubiquitinated forms of the Na,K-ATPase β-subunit in A549 cells exposed to elevated CO_2_ levels, suggesting that hypercapnia promoted the ubiquitination of the Na,K-ATPase β-subunit at the plasma membrane (Figure 2B). To identify the specific lysine (Lys) residue(s) in the Na,K-ATPase β-subunit that serve(s) as ubiquitin acceptor(s), A549 cells were transfected with V5-β1 variants in which we introduced Lys to Arg substitutions at each Lys in the intracellular domain of the Na,K-ATPase β-subunit. After exposure of cells to hypercapnia for 15 min, V5-β1 wt or mutants were immunoprecipitated and the ubiquitinated Na,K-ATPase β-subunit forms were detected with an anti-ubiquitin antibody. Replacement of Lys^5^ and Lys^7^ with Arg (V5-β1 K5/7R), led to a severe blunting of hypercapnia-induced Na,K-ATPase β-subunit ubiquitination (Figure 2C). Importantly, when Lys^5^ and Lys^7^ of the Na,K-ATPase β-subunit were simultaneously replaced with Arg the hypercapnia-induced endocytosis of the Na,K-ATPase β-subunit was also prevented (Figure 2D). Altogether, these results demonstrate that ubiquitination of the Na,K-ATPase β-subunit is a critical mediator of Na,K-ATPase β-subunit endocytosis. In similar experiments, we confirmed that the hypercapnia-induced endocytosis of the Na,K-ATPase β-subunit was not prevented when the remaining intracellular Lys residues of the Na,K-ATPase β-subunit were substituted (variants K13/14R and K21/22R) (Figure 2E) or when Lys^5^ and Lys^7^ were separately replaced by Arg (variants K5R and K7R) (Figure 2F). These findings raise the question of whether ubiquitin molecules are attached to both Lys^5^ and Lys^7^ simultaneously, or to one or the other Lys residue. Alternatively, it is possible that there is a preferred Lys residue, but when mutated, the other Lys plays a compensatory role. The flexibility in the ubiquitination site is not surprising, since the reactivity of a Lys is enhanced by the presence of basic residues in the vicinity of the E3 ligase binding site (Beal et al., 1996; Yamano et al., 1998; Deshaies and Joazeiro, 2009).

### Phosphorylation at Ser^11^ by PKC-ζ Is Required for the Hypercapnia-Induced, Ubiquitination-Driven Endocytosis of the Na,K-ATPase β-Subunit

**FIGURE 3:**
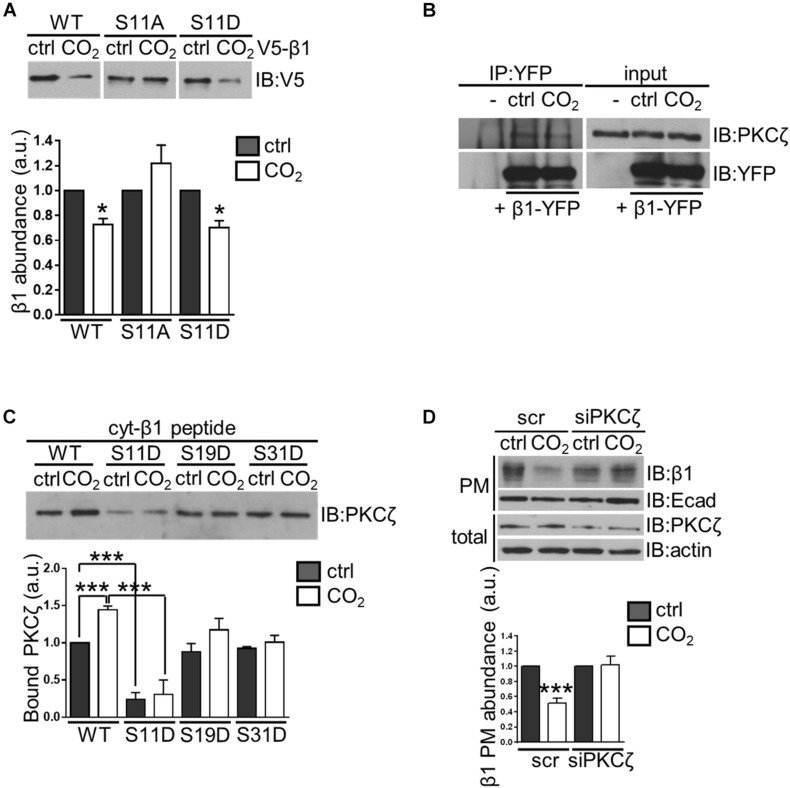
Phosphorylation of the Na,K-ATPase β-subunit at serine 11 by PKC-ζ is required for hypercapnia-induced ubiquitination-driven endocytosis of the protein in alveolar epithelial cells. **(A)** A549 cells were transfected with V5- β1 wt, V5- β1 S11A or V5- β1 S11D and surface proteins were labeled with cell-impermeable biotin and chased in 40 or 110 mmHg CO_2_ at a pH of 7.4 for 1 h. Afterward, streptavidin pull-down was performed and Na,K-ATPase β-subunit abundance was analyzed by IB. Mean ± SEM, *n* = 3, paired *t*-test, *p < 0.05. **(B)** A549-β1-YFP cells were exposed to 40 or 110 mmHg CO_2_ at a pH of 7.4 for 10 min. β1-YFP protein was immunoprecipitated from total lysates with an anti-YFP monoclonal antibody and PKC-ζ was detected with an anti-PKC-ζ monoclonal antibody by IB. Loading was controlled with an anti-YFP monoclonal antibody **(C)** A549 cells were exposed to 40 or 110 mmHg CO_2_ at a pH of 7.4 for 10 min. Total lysates were incubated with biotinylated synthetic peptides containing the wt or mutated intracellular domain of the Na,K-ATPase β-subunit (cyt-β1 peptide wt, S11D, S19D, and S31D) overnight and streptavidin pull-downs were performed. PKC-ζ was detected with an anti-PKC-ζ monoclonal antibody by IB. Mean ± SEM, *n* = 3, one-way ANOVA and Tukey’s *post hoc* test for multiple comparisons, ***p < 0.0005 (β1 wt Ctrl vs β1 wt CO_2_ and β1 wt vs β1 S11D). **(D)** A549 cells were transfected with siRNA against PKC-ζ or scrambled siRNA. 72 h after transfection, cells were exposed to 40 or 110 mmHg CO_2_ at a pH of 7.4 for 30 min. Biotin-streptavidin pull-down of cell surface proteins was performed and Na,K-ATPase β-subunit abundance was analyzed by IB. Total protein lysates are also shown. Bars represent mean ± SEM, *n* = 3, paired *t*-test, *p < 0.05.

Protein phosphorylation at amino acid residues that are in close proximity to the sites of ubiquitination is often required for protein ubiquitination and subsequent degradation (Deshaies, 1997). Based on proximity, the Ser^11^ residue of the Na,K-ATPase β-subunit was a potential phosphorylation site. To test this hypothesis, A549 cells were transfected with V5-β1 constructs in which Ser^11^ to Ala and Ser^11^ to Asp substitutions were introduced (S11A and S11D, respectively). Indeed, the hypercapnia-induced Na,K-ATPase β-subunit degradation was prevented in the S11A variant but not in the S11D variant (Figure 3A). These findings suggest that phosphorylation of Ser^11^ may be required for hypercapnia-induced ubiquitination and degradation of the Na,K-ATPase β-subunit.

The serine/threonine kinase protein kinase C (PKC)-ζ, which is activated in response to elevated CO_2_ levels and translocates to the plasma membrane (Briva et al., 2007; Vadasz et al., 2008), is a potential mediator of hypercapnia-induced phosphorylation of the Na,K-ATPase β-subunit. We first examined whether PKC-ζ interacts with the Na,K-ATPase β-subunit. To this end, A549 cells stably expressing the Na,K-ATPase β-subunit that was fused to an intracellular yellow fluorescent protein (YFP) tag (A549-β1-YFP) were used in co-immunoprecipitation studies. After exposing A549-β1-YFP cells to normocapnia or hypercapnia for 10 min, the β1-YFP protein was immunoprecipitated from total protein lysates. These experiments revealed that PKC-ζ co-immunoprecipitated with the Na,K-ATPase β-subunit under both normocapnic and hypercapnic conditions, indicating an interaction between these two proteins (Figure 3B). To confirm the binding of PKC-ζ to the Na,K-ATPase β-subunit, and to further verify the phosphorylation site in the Na,K-ATPase β-subunit, we conducted *in vitro* experiments in which we designed synthetic peptides containing the entire intracellular domain of the Na,K-ATPase β-subunit (cyt-β1 peptide). We mutated each Ser residue of the Na,K-ATPase β-subunit to Asp to mimic phosphorylation, and identified which variant affected PKC-ζ interaction with the Na,K-ATPase β-subunit. Protein extracts from A549 cells exposed to normocapnia or hypercapnia for 10 min were incubated with biotinylated cyt-β1 peptide (wt, S11D, S19D, and S31D) followed by streptavidin pull-downs. These experiments revealed that elevated CO_2_ levels led to an increase in PKC-ζ interaction with the Na,K-ATPase β-subunit and only the Ser^11^ to Asp variant prevented PKC-ζ binding to the Na,K-ATPase β-subunit (Figure 3C). To determine whether PKC-ζ was required for the hypercapnia-induced endocytosis of the Na,K-ATPase β-subunit, Na,K-ATPase β-subunit abundance in the plasma membrane was assessed in hypercapnia-exposed A549 cells in which PKC-ζ was depleted. In these studies, A549 cells were transfected with an siRNA directed against PKC-ζ or a scrambled siRNA, and exposed to normocapnia or hypercapnia for 30 min. In biotin-streptavidin pull-down experiments, we observed that PKC-ζ knockdown prevented the hypercapnia-induced endocytosis of the Na,K-ATPase β-subunit (Figure 3D). Furthermore, biotin-streptavidin pull-down experiments in the presence of the PKC inhibitor bisindolmaleimide I (Bis) demonstrated that inhibition of PKC activity prevented the hypercapnia-induced endocytosis of the Na,K-ATPase β-subunit (Supplementary Figure 2). Taken together, these results provide strong evidence that activity of PKC-ζ, phosphorylating the Na,K-ATPase β-subunit at Ser^11^ is required for hypercapnia-induced ubiquitination and endocytosis. Since PKC-ζ has also been documented to mediate CO_2_ effects on the Na,K-ATPase α-subunit, promoting the endocytosis of the protein (Briva et al., 2007; Vadasz et al., 2008), further studies will be required to evaluate whether the Na,K-ATPase α- and β-subunits are regulated independently or as a heterodimer. However, it is well established that the Na,K-ATPase α- and β-subunits are assembled in the endoplasmic reticulum in a 1:1 stoichiometric ratio (Tokhtaeva et al., 2009; Kryvenko et al., 2021) and are present at equimolar amounts at the plasma membrane (Craig and Kyte, 1980; Peterson and Hokin, 1981; Brotherus et al., 1983). Most interestingly, PKC-ζ directly phosphorylates both the Na,K-ATPase α- and β-subunits. These phosphorylation events are necessary for initiation of the hypercapnia-induced endocytosis of the Na,K-ATPase α/β-complex, which is mediated by JNK, LMO7b, and α-adducin (Vadasz et al., 2012a; Lecuona et al., 2013; Dada et al., 2015). Together, these findings support the notion that the Na,K-ATPase α- and β-subunits are probably regulated as a dimer in the setting of hypercapnia.

### TRAF2 Mediates the Hypercapnia-Induced Ubiquitination of the Na,K-ATPase β-Subunit

**FIGURE 4:**
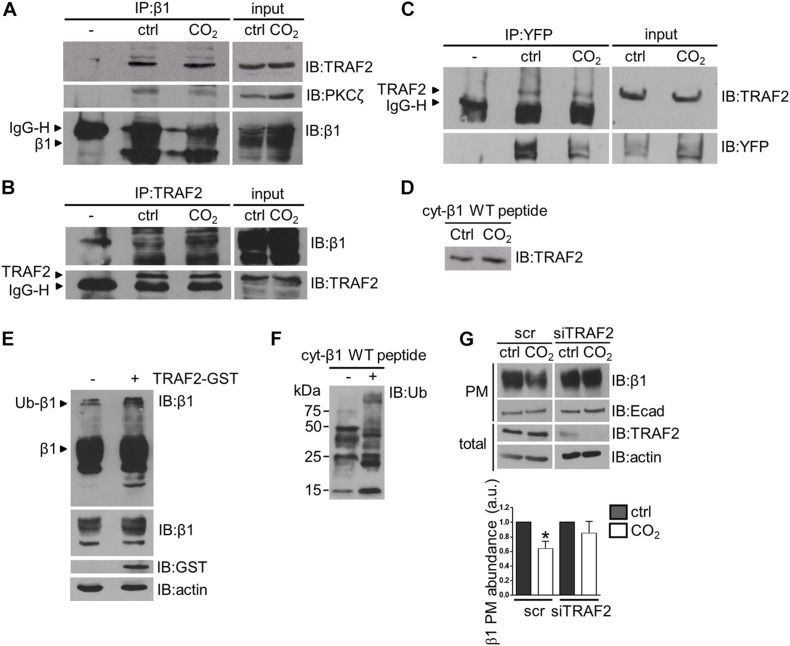
TRAF2 mediates the hypercapnia-induced ubiquitination of the Na,K-ATPase β-subunit in alveolar epithelial cells. **(A)** A549 were exposed to 40 or 110 mmHg CO_2_ at a pH of 7.4 for 10 min. Na,K-ATPase β-subunit was immunoprecipitated from total lysates with anti-Na,K-ATPase β1-subunit monoclonal antibody and TRAF2 and PKC-ζ were detected with their respective antibodies by IB. Loading was controlled with an anti-Na,K-ATPase β1-subunit monoclonal antibody. Representative western blots are shown. **(B)** A549 cells were exposed to 40 or 110 mmHg CO_2_ at a pH of 7.4 for 10 min. TRAF2 was immunoprecipitated from total lysates with anti-TRAF2 polyclonal antibody and Na,K-ATPase β-subunit was detected with an anti-Na,K-ATPase β1-subunit antibody by IB. Loading was controlled with an anti-TRAF2 monoclonal antibody. Representative western blots are shown. **(C)** A549-β1-YFP cells were exposed to 40 or 110 mmHg CO_2_ at a pH of 7.4 for 10 min. β1-YFP protein was immunoprecipitated from total lysates with an anti-YFP monoclonal antibody and TRAF2 was detected with an anti-TRAF2 monoclonal antibody by IB. Loading was controlled with anti-YFP monoclonal antibody. **(D)** A549 cells were exposed to 40 or 110 mmHg CO_2_ at a pH of 7.4 for 10 min. Total lysates were incubated with the biotinylated cyt-β1 wt peptide overnight and streptavidin pull-downs were performed. TRAF2 was detected with anti-TRAF2 monoclonal antibody by IB. **(E)** Ubiquitinated Na,K-ATPase β-subunit forms were detected in total lysates from A549 cells transfected with TRAF2-GST construct or untransfected, by an anti-Na,K-ATPase β-subunit antibody. Overexposure of the x-ray films revealed Na,K-ATPase β-subunit polyubiquitinated forms. **(F)** TRAF2 IP from A549 cells was used to ubiquitinate cyt-β1 wt peptide in an *in vitro* ubiquitination reaction. As a negative control the cyt-β1 wt peptide was omitted. Biotinylated cyt-β1 wt peptides were purified by streptavidin pull-downs and the Na,K-ATPase β-subunit ubiquitinated forms were detected with an anti-ubiquitin antibody by IB. **(G)** A549 cells were transfected with siRNA against TRAF2 or scrambled siRNA. 48 h after transfection, cells were exposed to 40 or 110 mmHg CO_2_ at a pH of 7.4 for 30 min. Biotin-streptavidin pull-down of cell surface proteins was performed and Na,K-ATPase β-subunit abundance was analyzed by IB. Mean ± SEM, *n* = 3, paired *t*-test, *p < 0.05.

Our data suggest that elevated CO_2_ levels lead to downregulation of Na,K-ATPase β-subunit plasma membrane abundance by ubiquitination of the protein at Lys^5^ and Lys^7^, which acts as a signal for protein endocytosis. In the ubiquitination process, substrate specificity is dictated by E3 ubiquitin ligases (Metzger et al., 2012). Therefore, we set out to identify the E3 ligase involved in the hypercapnia-induced Na,K-ATPase β-subunit ubiquitination. To this end, we applied a protein microarray to screen for proteins that interact with the human Na,K-ATPase β-subunit. Protein lysates from cells expressing a phosphomimic mutant of the Na,K-ATPase β-subunit (V5-β1 S11/31D) were applied to a microarray of more than 9,000 human proteins, among which 239 were ubiquitin E3 ligases. The results of this microarray are presented in the Supplementary Material. After further analysis of the E3 ligases that were found to interact with the Na,K-ATPase β-subunit in the protein microarray, we identified TRAF2 as a potential candidate. TRAF2 is a RING E3 ligase, probably best known for its role in the regulation of the NF-κB signaling pathway (Alvarez et al., 2010). TRAF2 catalytic activity has been shown to be promoted by the binding of sphingosine-1-phosphate (S1P), intracellularly produced by sphingosine kinase 1 (Sphk1) (Alvarez et al., 2010). Three reasons led us to consider TRAF2 as a potential E3 ligase for the Na,K-ATPase β-subunit. First, we found a major TRAF2 binding motif (P/S/T/A)X(Q/E)E (Ye et al., 1999) in the Na,K-ATPase β-subunit intracellular domain, which comprises Lys^7^ and is in close proximity to Lys^5^ and Ser^11^ of the Na,K-ATPase β-subunit. Second, sphingosine kinase 2 (Sphk2) was also found to significantly interact with the Na,K-ATPase β-subunit in the protein microarray, and we speculated that S1P production by Sphk2, in proximity to the Na,K-ATPase β-subunit may serve as the cofactor required for TRAF2 ubiquitin ligase activity. Third, TRAF2 mediates K63-linked polyubiquitination (Alvarez et al., 2010), which has been shown to regulate the endocytosis and protein sorting of some plasma membrane proteins (Alvarez et al., 2010) and could therefore regulate the ubiquitination-driven endocytosis of the Na,K-ATPase β-subunit under hypercapnic conditions. The interaction between endogenous TRAF2 and Na,K-ATPase β-subunit was confirmed by co-immunoprecipitation studies. Cells were exposed to normocapnia or hypercapnia for 10 min and the Na,K-ATPase β-subunit was immunoprecipitated from total protein lysates. These experiments revealed that TRAF2 co-immunoprecipitated with Na,K-ATPase β-subunit under both normocapnic and hypercapnic conditions (Figure 4A). The reverse co-immunoprecipitation confirmed the interaction between the Na,K-ATPase β-subunit and TRAF2 (Figure 4B). Of note, we were also able to detect PKC-ζ in these co-immunoprecipitated protein complexes (Figure 4A). The binding of TRAF2 to the Na,K-ATPase β-subunit was also confirmed in co-immunoprecipitation studies in A549-β1-YFP cells (Figure 4C). The TRAF2 interaction with the Na,K-ATPase β-subunit was also detected in *in vitro* studies, in which total lysates from A549 cells exposed to normocapnia or hypercapnia for 10 min were incubated with biotinylated cyt-β1 wt peptides (Figure 4D). TRAF2-GST overexpression in A549 cells caused a substantial accumulation of the ubiquitinated Na,K-ATPase β-subunit forms (Figure 4E). To evaluate whether TRAF2 directly mediates ubiquitination of the Na,K-ATPase β-subunit, we conducted *in vitro* ubiquitination experiments, in which immunoprecipitated TRAF2 supplemented with its the cofactor, S1P, were employed as a source of E3 ubiquitin ligase to ubiquitinate the cyt-β1 wt peptide. Following the ubiquitination reaction, biotinylated cyt-β1 wt peptides were isolated by streptavidin pull-down, and the ubiquitinated Na,K-ATPase β-subunit forms were detected with anti-ubiquitin antibodies. In these studies, we observed a significant accumulation of ubiquitinated Na,K-ATPase β-subunit forms (Figure 4F). To determine whether TRAF2 was required for the hypercapnia-induced endocytosis of the Na,K-ATPase β-subunit, we determined the plasma membrane abundance of the Na,K-ATPase β-subunit in hypercapnia-exposed A549 cells in which TRAF2 was depleted using specific siRNA. Results from these experiments revealed that TRAF2 depletion was sufficient to prevent the hypercapnia-induced endocytosis of the Na,K-ATPase β-subunit (Figure 4G). Together, these results provide evidence that TRAF2 acts as the E3 ligase for the Na,K-ATPase β-subunit, mediating the effects of hypercapnia on Na,K-ATPase β-subunit stability.

Of note, the experiments in which TRAF2 protein was depleted from cells did not exhibit an increase in total plasma membrane abundance of Na,K-ATPase β-subunit protein. One possible explanation for this observation is that TRAF2 may not be involved in the turnover of the Na,K-ATPase β-subunit under steady-state conditions. Although required for the hypercapnia-induced ubiquitination of the Na,K-ATPase β-subunit, TRAF2 may not be the only E3 ligase involved in the regulation of surface expression of the Na,K-ATPase β-subunit. Moreover, ubiquitination might not be the only mechanism involved in the endocytosis of the Na,K-ATPase β-subunit. Other mechanisms may account for Na,K-ATPase β-subunit endocytosis under normal or other stress-associated conditions. Furthermore, considering the role of ubiquitination in the protein degradation pathways (Ciechanover et al., 1984; Finley, 2009), it still remains to be elucidated whether the hypercapnia-induced β-subunit degradation requires other ubiquitination events involving other E3 ligases.

### Overexpression of the K5/7R Na,K-ATPase β-Subunit Restores Impaired Alveolar Epithelial Cell-Cell Junction Formation Upon Hypercapnia

**FIGURE 5:**
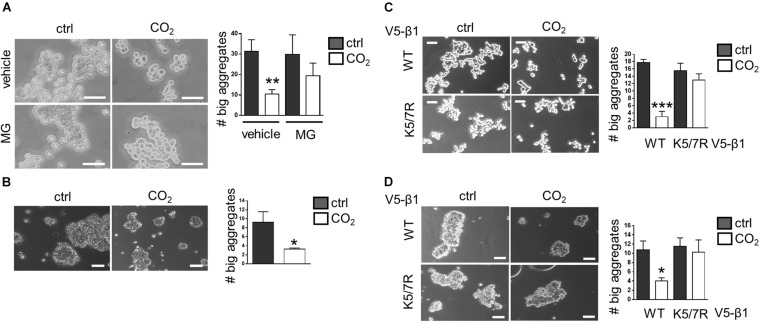
Expression of the K5/7R Na,K-ATPase β-subunit restores hypercapnia-impaired alveolar epithelial cell-cell junction formation. **(A)** A549 cells were exposed to 40 or 110 mmHg CO_2_ at a pH of 7.4 for 8 h. The number of aggregates containing more than 10 cells was determined. Mean ± SEM, *n* = 4, unpaired *t*-test, **p < 0.005. Scale bar = 50 μM. **(B)** A549 cells were exposed to 40 or 110 mmHg CO_2_ at a pH of 7.4 for 4 h. The number of aggregates containing more than 20 cells was determined. Mean ± SEM, *n* = 3, unpaired *t*-test, **p < 0.005. Scale bar = 50 μM. **(C)** A549 nucleofected with V5-β1 wt or V5-β1 K5/7R were exposed to 40 or 110 mmHg CO_2_ at a pH of 7.4 for 4 h. The number of aggregates containing more than 20 cells was determined. Mean ± SEM, *n* = 3, unpaired *t*-test, ***p < 0.0005. Scale bar = 50 μM. **(D)** Rat primary ATII cells nucleofected with V5-β1 wt or V5-β1 K5/7R were exposed to 40 or 110 mmHg CO_2_ at a pH of 7.4 for 4 h. The number of aggregates containing more than 20 cells was determined. Mean ± SEM, *n* = 3, unpaired *t*-test, ***p < 0.0005. Scale bar = 10 μM.

To evaluate whether elevated CO_2_ levels affect the ability of alveolar epithelial cells to interact with each other we conducted a cell aggregation assay in which a single-cell suspension of A549 cells was incubated under normocapnic or hypercapnic conditions for 8 h. Data analysis revealed that the number of large aggregates, defined as aggregates containing more than 10 cells, was significantly reduced in hypercapnia compared to normocapnia, suggesting that elevated CO_2_ levels were impairing the ability of cells to form junctions (Figure 5A). These results were confirmed in a second set of studies conducted in rat primary alveolar epithelial type II (ATII) cells. Due to the highly-adhesive nature of these cells, primary ATII cells formed larger aggregates than A549 cells. However, similar to A549 cells, the adhesive properties of ATII cells were impaired in cells exposed to hypercapnia for 4 h, thus demonstrating the adverse effects of elevated CO_2_ levels on the ability of cells to establish cell-cell contacts (Figure 5B). To demonstrate a causal link between CO_2_-mediated effects on plasma membrane Na,K-ATPase β-subunit stability and cell-cell adhesion, we expressed a V5-β1 K5/7R variant, which remains at the cell surface under hypercapnic conditions, and assessed the capacity of these cells to restore formation of cell-cell junctions after exposure to elevated CO_2_ levels. Results from cell aggregation assays in which A549 cells were nucleofected with V5-β1 wt or K5/7R variant indicated that while hypercapnia inhibited the formation of larger aggregates in cells expressing V5-β1 wt, this behavior was prevented in cells expressing V5-β1 K5/7R, in which no differences in the aggregate sizes were found between normocapnia- and hypercapnia-treated cells (Figure 5C). The above-mentioned results were confirmed in a second set of experiments conducted in rat primary ATII cells nucleofected with V5-β1 wt or K5/7R variants, a novel transfection methodology for hard-to-transfect cell types, which is well established in our laboratory (Grzesik et al., 2013). Protein expression of V5-β1 wt and V5-β1K5/7R proteins was confirmed by SDS-PAGE followed by western blot (Supplementary Figure 3). Similar to A549 cells, impairment of cell junction formation in hypercapnia-exposed cells was prevented in K5/7R variants, thereby demonstrating that the ubiquitination-driven endocytosis of the Na,K-ATPase β-subunit was indeed the underlying mechanism by which hypercapnia impaired the formation of cell junction (Figure 5D).

**FIGURE 6:**
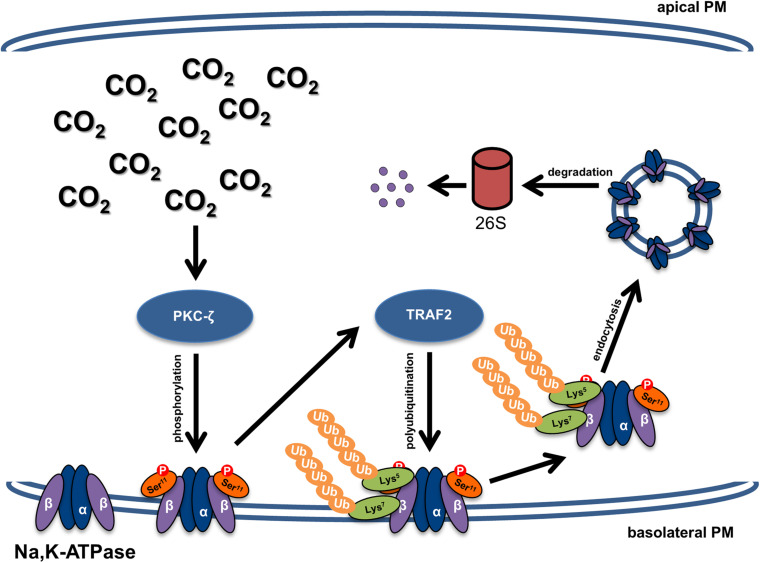
Schematic representation of the mechanism of hypercapnia-induced downregulation of the Na,K-ATPase β-subunit and impaired alveolar epithelial barrier integrity. Elevated CO_2_ levels lead to activation of PKC-ζ, which phosphorylates the Na,K-ATPase β-subunit at serine 11, thereby making the protein a target of polyubiquitination at lysine residues 5 and 7 by TRAF2. Polyubiquitination of the Na,K-ATPase β-subunit promotes internalization of the protein from the plasma membrane and subsequent proteasomal degradation, which impairs the junctional function of the Na,K-ATPase β-subunit and thus leads to alveolar epithelial barrier dysfunction.

In the present work we elucidated the molecular mechanism involved in hypercapnia-induced downregulation of the Na,K-ATPase β-subunit (Figure 6). We demonstrated that elevated CO_2_ levels lead to PKC-ζ-mediated phosphorylation of the Na,K-ATPase β-subunit, which triggers TRAF2-mediated ubiquitination, followed by endocytosis and degradation of the Na,K-ATPase β-subunit. The decision to orient our research toward the identification of the E3 ligase that drives endocytosis and degradation of the Na,K-ATPase β-subunit in the setting of hypercapnia was based on the notion that E3 ubiquitin ligases determine the specificity of the ubiquitination process by targeting specific substrates. Considering the relevant role of Na,K-ATPase β-subunit in cell adherens junction formation in alveolar epithelial cells, the identification of TRAF2 as the Na,K-ATPase β-subunit E3 ligase provides a potential therapeutic tool to be employed in lung diseases in which integrity of the alveolar epithelium is impaired, such as ARDS, lung fibrosis, chronic obstructive pulmonary disease or lung cancer.

## Data Availability Statement

The raw data supporting the conclusions of this article will be made available by the authors, without undue reservation.

## Ethics Statement

The animal study was reviewed and approved by the local authorities (Regierungspräsidium Giessen) in Germany.

## Author Contributions

NG and IV designed the studies and drafted the manuscript. NG, LM, and VK executed the studies. NG, LM, VK, KT, SH, RM, FG, LD, WS, JS, and IV analyzed and interpreted the results. All authors contributed significant edits, gave final approval for publication, and agreed to be accountable for the integrity of the information contained in this manuscript.

## Conflict of Interest

The authors declare that the research was conducted in the absence of any commercial or financial relationships that could be construed as a potential conflict of interest.
