# Supplementary material for: TRAF2 Is a Novel Ubiquitin E3 Ligase for the Na,K-ATPase β-Subunit That Drives Alveolar Epithelial Dysfunction in Hypercapnia
Source: Front Cell Dev Biol. 2021 Jul 2;9:689983. doi: 10.3389/fcell.2021.689983 (PMC8283768; doi:10.3389/fcell.2021.689983)
Supplement: Supplementary file 1 [file Data_Sheet_1.docx]

**SUPPLEMENTARY MATERIAL**

TRAF2 is a novel ubiquitin E3 ligase for the Na,K-ATPase β‑subunit that drives alveolar epithelial barrier dysfunction in hypercapnia

**Nieves M. Gabrielli^1^, Luciana C. Mazzocchi^1^, Vitalii Kryvenko^1,2^, Khodr Tello^1,2^, Susanne Herold^1,2^, Rory E. Morty^1,2,3^, Friedrich Grimminger^1,2,4^, Laura A. Dada^5^, Werner Seeger^1,2,3,4^, Jacob I. Sznajder^5^, and István Vadász^1,2*^**

^1^Department of Internal Medicine, Justus Liebig University, Universities of Giessen and Marburg Lung Center (UGMLC), Member of the German Center for Lung Research (DZL), 35392 Giessen, Germany;

^2^The Cardio-Pulmonary Institute (CPI), 35392 Giessen, Germany

^3^Department of Lung Development and Remodelling, Max Planck Institute for Heart and Lung Research, 61231 Bad Nauheim, Germany;

^4^Institute for Lung Health (ILH), 35392 Giessen, Germany

^5^Division of Pulmonary and Critical Care Medicine, Feinberg School of Medicine, Northwestern University, Chicago, IL 60611, USA

*** Correspondence:**István Vadász, MD, PhD
[istvan.vadasz@innere.med.uni-giessen.de](mailto:istvan.vadasz@innere.med.uni-giessen.de)

**FIGURE LEGENDS**

**Supplementary Figure 1:** (*A*) A549 cells were transfected with V5-β1 wt and exposed to 40 or 110 mmHg CO_2_ at a pH of 7.4 for 30 min. Total protein lysates were analyzed by IB. Representative blots showing the expression of V5-β1 are displayed. (*B*) A549 cells were exposed to 40 or 110 mmHg CO_2_ at a pH of 7.4 for 30 min. Total protein lysates were analyzed by IB. A representative blot showing the expression of Na,K-ATPase β1-subunit is depicted.

**Supplementary Figure 2:** A549 cells were pre-incubated with vehicle (DMSO) or the PKC inhibitor, bisindolmaleimide I (Bis) for 30 min and exposed to 40 or 110 mmHg CO_2_ at a pH of 7.4 for 30 min. Biotin-streptavidin pull-down of cell surface proteins was performed and analyzed by IB. Mean ± SEM, n = 3, paired t-test, *p<0.05.

**Supplementary Figure 3:** (*A*) A549 cells were nucleofected with V5-β1 wt or V5-β1 K5/7R. Total protein lysates were analyzed by IB 18 h after transfection. (*B*) ATII cells were nucleofected with V5-β1 wt or V5-β1 K5/7R. Total protein lysates were analyzed by IB 48 h after transfection.

**Supplementary Tables**

**Table S1. Amino acid sequence of biotinylated synthetic peptides**

| **Construct** | **Peptide** |
| --- | --- |
| **WT** | Biotin-MARGKAKEEGSWKKFIWNSEKKEFLGRTGGSWFK |
| **S11D** | Biotin-MARGKAKEEGDWKKFIWNSEKKEFLGRTGGSWFK |
| **S19D** | Biotin-MARGKAKEEGSWKKFIWNDEKKEFLGRTGGSWFK |
| **S31D** | Biotin-MARGKAKEEGSWKKFIWNSEKKEFLGRTGGDWFK |

**Table S2. Nucleotide sequence of primers employed for cloning and site-directed mutagenesis**

| **Construct** | **Primer (5´to 3´)** |
| --- | --- |
| **WT** | Forward: 5´ ATAGGATCCGTAATGGCCCGCGGGAAAGCCAAG 3´  Reverse: 5´ CTGGAATTCGCTCTTAACTTCAATTTTTACATC 3´ |
| **K5RK7R** | 5´GGATCCGTAATGGCCCGCGGGAGAGCCAGGGAGGAGGGCAGCTGG3´ |
| **K13RK14R** | 5´GAGGAGGGCAGCTGGAGGAGATTCATCTGGAACTCAG3´ |
| **K21RK22R** | 5´CATCTGGAACTCAGAGAGGAGGGAGTTTCTGGGCAG3´ |
| **S11A** | 5´GCCCGCGGGAAAGCCAAGGAGGAGGGCGCCTGGAAGAAATTCATCTGG3´ |
| **S11D** | 5´GCCCGCGGGAAAGCCAAGGAGGAGGGCGACTGGAAGAAATTCATCTGG3´ |
