# Supplementary figures and images for: TRAF2 Is a Novel Ubiquitin E3 Ligase for the Na,K-ATPase β-Subunit That Drives Alveolar Epithelial Dysfunction in Hypercapnia
Source: Front Cell Dev Biol. 2021 Jul 2;9:689983. doi: 10.3389/fcell.2021.689983 (PMC8283768; doi:10.3389/fcell.2021.689983)

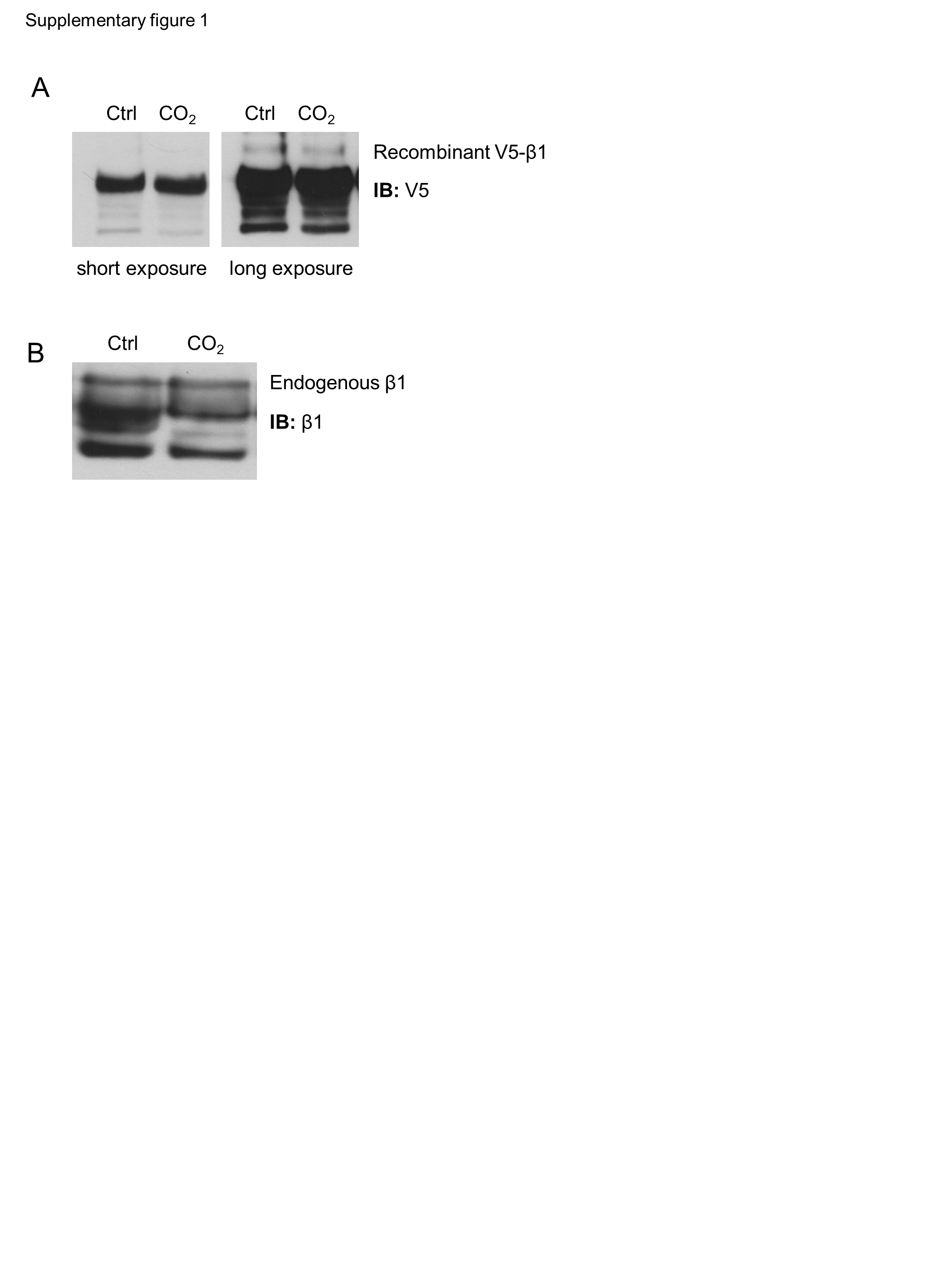

Supplement: Supplementary file 2 [file Image_1.TIF]

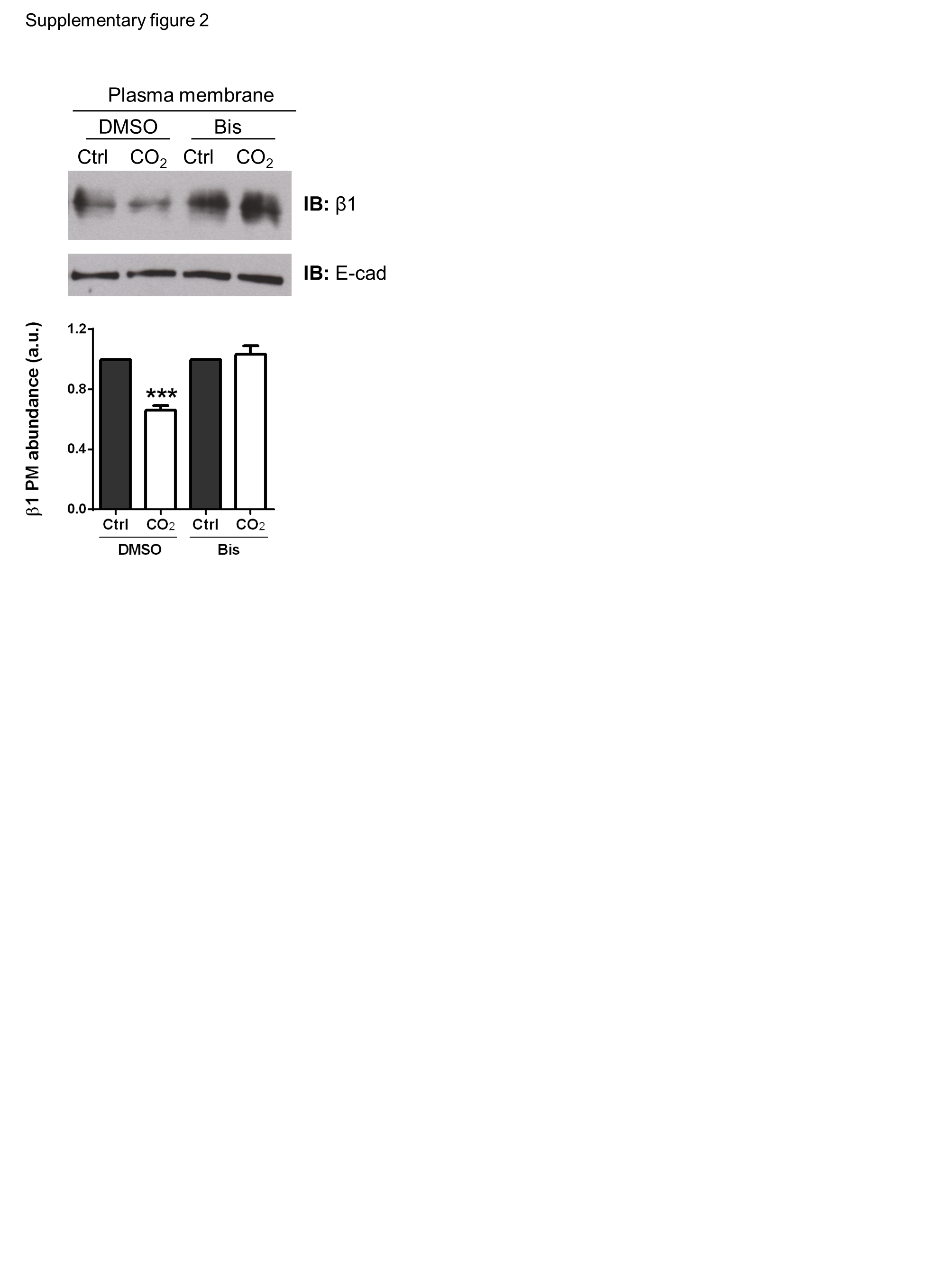

Supplement: Supplementary file 3 [file Image_2.TIF]

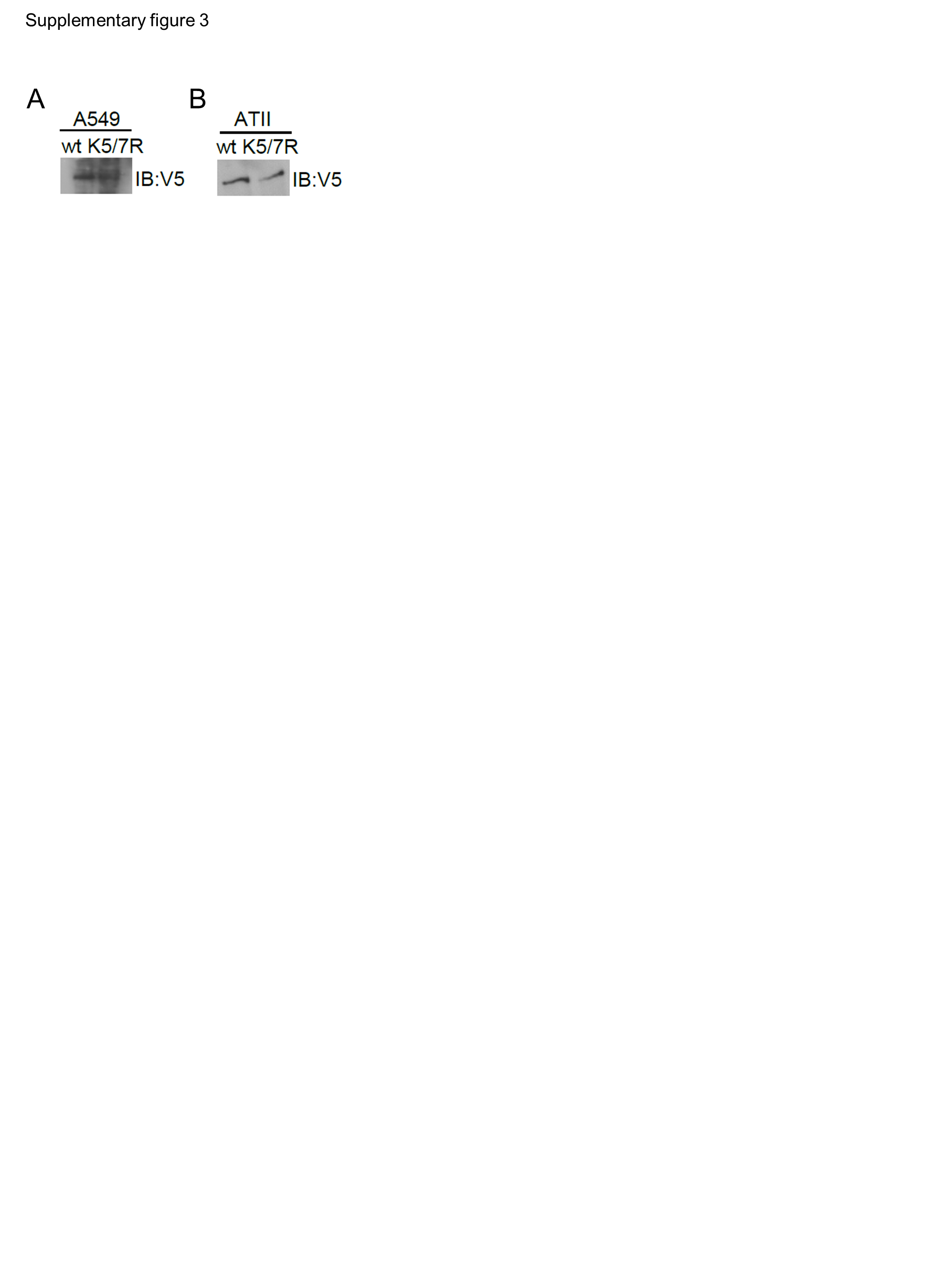

Supplement: Supplementary file 4 [file Image_3.TIF]
